# Assembly mechanisms, not species pool, shape *β*-diversity of soil methanotrophic communities in steppes of China

**DOI:** 10.3389/fmicb.2024.1522319

**Published:** 2025-01-20

**Authors:** Yongping Kou, Zhe Feng, Huan Li, Yanjiao Liu, Lin Xu, Xiangzhen Li

**Affiliations:** ^1^CAS Key Laboratory of Mountain Ecological Restoration and Bioresource Utilization & Ecological Restoration and Biodiversity Conservation Key Laboratory of Sichuan Province & China-Croatia "Belt and Road" Joint Laboratory on Biodiversity and Ecosystem Services, Chengdu Institute of Biology, Chinese Academy of Sciences, Chengdu, China; ^2^School of Public Health, Lanzhou University, Lanzhou, China; ^3^School of Earth System Science, Institute of Surface-Earth System Science, Tianjin University, Tianjin, China; ^4^National Forestry and Grassland Administration Key Laboratory of Forest Resources Conservation and Ecological Safety on the Upper Reaches of the Yangtze River & Forestry Ecological Engineering in the Upper Reaches of the Yangtze River Key Laboratory of Sichuan Province, Sichuan Agricultural University, Chengdu, China; ^5^Engineering Research Center of Soil Remediation, Fujian Province University, College of Resources and Environment, Fujian Agriculture and Forestry University, Fuzhou, China

**Keywords:** species pool, community assembly, deterministic processes, stochastic processes, soil methanotrophic communities

## Abstract

**Introduction:**

One of the central aims in ecology is elucidating the mechanisms that shape community diversity. While biodiversity patterns across geographical gradients are often attributed both to local assembly processes and regional species pools, the distinct roles of these factors in shaping soil aerobic methanotrophic diversity remain underexplored.

**Methods:**

Using amplicon sequencing and bioinformatics analysis, this study focuses on comparing the relative importance of species pool and community assembly processes in shaping soil methanotrophic communities across three distinct plateaus in China: the Loess Plateau, the Qinghai-Tibetan Plateau, and the Inner Mongolian Plateau. Each of these plateaus includes three distinct steppe habitats: desert, meadow, and typical steppe.

**Results:**

Our findings reveal that *pmoA* beta (*β*)-diversity followed a distance-decay pattern, which declined with geographical distance at different rates depending on the steppe type and area, potentially due to diverse mechanisms of community assembly. Moreover, a decoupling between *β*-diversity and gamma-diversity observed, suggesting that local community assembly mechanisms primarily account for variations in *β*-diversity patterns. Furthermore, the relative significance of these assembly processes (e.g., dispersal limitation, drift, environmental filtering, and biotic interactions) varies according to spatial scales and steppe types. Notably, the differential environmental conditions (such as soil pH, yearly average temperature, and precipitation) across scales and steppe habitats primarily modulate the intensity of these assembly processes, thereby influencing *β*-diversity.

**Conclusion:**

In summary, our study emphasizes the crucial role of local community assembly in changing soil methanotrophic *β*-diversity’s geographical patterns, highlighting the significance of a nuanced understanding of these processes for effective conservation and management strategies.

## 1 Introduction

Community *β*-diversity, which encompasses inter-site compositional discrepancies, serves as a powerful tool for deciphering cross-scale diversity patterns and inferring the processes that structure microbial communities. It enables effective bridging between local (*α-*) diversity and regional (*γ-*) diversity (also known as species pools) (Myers et al., 2013). The Distance-decay relationship (DDR), which describes the increase in community *β*-diversity with geographical distance, has been widely used to uncover directional changes in both macro and microbial communities (Morlon et al., 2008). DDR has also been applied to specific functional microbial communities, such as diazotrophs and alkaline phosphatase (*phoD*) gene-encoding bacterial communities (Xu et al., 2021, 2024). However, previous studies often focused on DDR slopes, leaving the underlying mechanisms driving these relationships largely unexplored.

Drawing from the modern coexistence theory of macroecological origin, the species pool and local community assembly mechanisms jointly shape community *β*-diversity patterns (Zhang et al., 2020). A larger species pool can provide richer candidate species for colonization in a particular habitat, resulting in stronger biotic interactions and potentially greater *β*-diversity (Xu et al., 2021). Deterministic processes involve niche-based and nonrandom mechanisms (Vellend and Agrawal, 2010), encompassing interspecific interactions and environmental filtering, which can impact *β*-diversity differently. For instance, environmental variables, such as climate, plant species, soil pH, and soil permeability (Li et al., 2024; Kou et al., 2021), have been found to mediate soil aerobic methane-oxidizing bacteria (aMOBs) community assembly, enabling selective species filtration from regional species pool by various means across local communities, leading to high *β*-diversity (Dini-Andreote et al., 2015). On the other hand, a homogeneous environment leads to low *β*-diversity through similar selective species filtration (Catano et al., 2017). Biotic interactions among species, often inferred using co-occurrence network analysis, also play a role in determining *β*-diversity (Xu et al., 2024). High intraspecific and low interspecific competition can foster high *β*-diversity, while the opposite results in low *β*-diversity (Barabas et al., 2016; Zhao et al., 2019). Stochastic processes mainly show random variations in relative species abundances, including stochastic birth-death and dispersal processes (Chase and Myers, 2011; Hubbell, 2005). Additionally, *β*-diversity fluctuations are probably dependent on the dispersal events as well (Chase and Myers, 2011; Hubbell, 2005). High dispersal rates including homogenizing dispersal (Stegen et al., 2012), may reduce *β*-diversity along with comprehensive species diversity through elevation of the prevailing species distributions (Albright and Martiny, 2018; Cadotte, 2006), whereas dispersal restriction would lead to the aggregation of spatial species distributions (Questad and Foster, 2008), increasing *β*-diversity across locations. Random birth and death events can result in either high or low *β*-diversity, depending on the intensity and frequency of these stochastic processes. However, the relative significance of the species pool and local community assembly mechanisms varies significantly among different taxonomic groups and habitats, shaped by unique environmental preferences (Li et al., 2022; Logue et al., 2015; Xu et al., 2021, 2024; Wang et al., 2021; Zhang et al., 2020). Thereby, our current understanding of the relative importance of species pool and local community assembly processes on community *β*-diversity pattern is far from complete, particularly regarding specific functional microbial communities. For example, it remains largely unknown about which mechanism play a more pivotal role in shaping soil aMOBs, a key functional group responsible for methane (CH_4_) oxidation.

The aMOBs in soil exert a pivotal role in mitigating greenhouse gas emissions by consuming methane CH_4_, the most prevalent greenhouse gas trailing only carbon dioxide (Tveit et al., 2019). These microorganisms annually consume approximately 30 Tg of CH_4_, making them the second major sink of atmospheric CH_4_ (Deng et al., 2019). The *pmoA*-gene is widely used in charactering the functional aMOBs (Schnyder et al., 2018). The importance of this functional community in CH₄ oxidation has been well established through studies conducted in Chinese steppes (Li et al., 2024; Kou et al., 2021). However, these investigations primarily addressed the community’s composition and its assembly processes. Two critical knowledge gap remains: firstly, there is a lack of understanding the DDR patterns of soil aMOB communities across different scales; secondly, whether the species pool (regional diversity) or local community assembly processes (e.g., environmental filtering) play a more significant role in shaping this functional community. Addressing this question is vital for understanding the ecological mechanisms underlying CH₄ oxidation in these ecosystems. This significant gap significantly constrains our comprehension of the formation and maintenance of terrestrial microbial community diversity.

In this study, we conducted a comprehensive investigation across three distinct temperature-precipitation transects located in the Loess (LP), Qinghai-Tibet (QTP) and Inner Mongolia (IMG) regions. Focusing on soil aMOB, which are notably featured by the *pmoA* gene encoding the particulate methane monooxygenase *β*-subunit (Fang et al., 2022), we were aimed at deciphering the potential driving mechanisms of *β*-diversity patterns for these aMOB at various biogeographical scales. Our objectives encompass: (1) Examining the biogeographical patterns of *pmoA β*-diversity, both collectively across the entire regions and individually within each habitat type and region; (2) Assessing the mediator roles played by the environmental filtration, species pool, ecological drift, dispersal events and biotic interactions among *pmoA* species in the *pmoA β*-diversity. (3) Identifying the key factors that govern the community assembly processes and *β*-diversity.

## 2 Materials and methods

### 2.1 Site description and soil sampling

**Figure 1:**
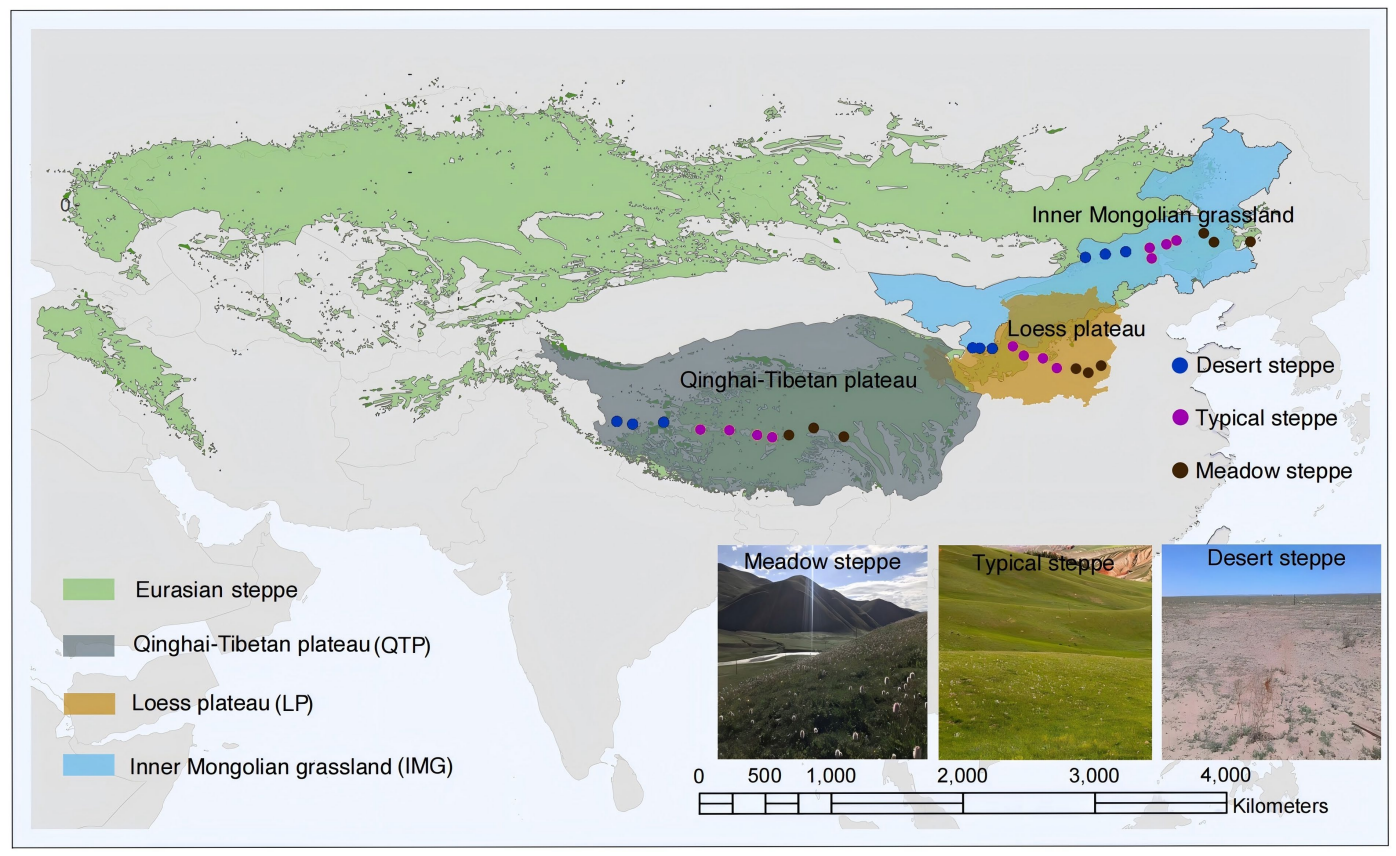
Geographical distributions and limiting factors of Loess plateau (LP), Inner Mongolian grassland (IMG) and Qinghai-Tibetan plateau (QTP).

Three distinct transects were established, each traversing the Loess (latitudes 35.99°N to 37.44°N and longitudes 104.92°E to 113.36°E), Inner Mongolia (spanning latitudes 43.63°N to 45.11°N and longitudes 112.15°E to 123.51°E) and Qinghai-Tibet regions (latitudes 31.38°N to 32.48°N and longitudes 80.15°E to 95.45°E), respectively (Figure 1; Supplementary Table S1). These transects, oriented east–west, encompassed precipitation-temperature gradients, measuring 800 km in Loess, 900 km in Inner Mongolia, and 1,500 km in Qinghai-Tibet, collectively spanning a distance of up to 4,000 km (Figure 1; Supplementary Table S1). Within each transect, ten undisturbed sampling sites were strategically positioned. To ensure representative sampling, a minimum distance of over 80 km between adjacent sites was setup (Xu et al., 2021). Within every region, we sampled soils from three steppe categories - meadow, desert and typical - each representing a unique habitat characterized by varying precipitation gradients. The meadow steppe features lush, highly productive vegetation under a subhumid climate; the typical steppe harbors drought-tolerant graminaceous plants under a semi-arid environment, while the desert steppe serves as a steppe-desert transitional zone, dominated by exceedingly xeric species like *Stipa* spp. Among the three regions, the Loess region boasts the highest mean annual temperature (MAT) at 9.13°C, followed by Inner Mongolia at 2.52°C and Qinghai-Tibet at −3.02°C (Supplementary Table S1). Similarly, mean annual precipitation (MAP) is highest in Loess (466.05 mm), followed by Qinghai-Tibet (428.84 mm) and Inner Mongolia (313.34 mm) (Supplementary Table S1). The aridity index (AI), quantifying precipitation availability over atmospheric water demand, was calculated dividing MAP by mean annual evapo-transpiration (Hargreaves, 1994; Title and Bemmels, 2018; Zorner et al., 2008), follows a similar trend, with Loess having the highest value (0.31), Qinghai-Tibet close behind (0.30), and Inner Mongolia the lowest (0.22) (Supplementary Table S1). Lastly, soil pH also varies, with Loess recording the highest level (8.06), followed by Inner Mongolia (7.96) and Qinghai-Tibet (7.81) (Supplementary Table S2).

Field sampling was carried out in the growing season, spanning from July to August 2018, across the three regions. Totally, among all sampling sites, 10, 10 and 10 sites are distributed in Loes, Inner Mongolia and Qinghai-Tibet, respectively. For representative sampling, each site was carefully selected based on several criteria. First, the site had to reflect the typical vegetation of the region and avoid atypical features, such as sandy patches in meadow steppe areas or sampling under stones or plants. Second, the site needed to be as far as possible from roads and free from cattle or sheep grazing to minimize human and livestock disturbance. This ensured the sampling captured natural conditions with minimal external influence. Within each sampling site, a designated 100 m × 100 m area served as the base for establishing four quadrats of 1 m × 1 m dimensions, ensuring a minimum distance of 10 meters between any two adjacent quadrats to promote randomness. Within each quadrat, a standardized protocol was followed to collect five random soil cores, each extending 0–10 cm in depth and 5 cm in diameter. Subsequently, these cores were combined in order to form a single composite sample, allowing for a comprehensive representation of the soil within that quadrat. Across all three regions, this rigorous sampling procedure resulted in the collection of a total of 120 soil samples. The collected samples were stored in a portable icebox with sufficient ice packs immediately after sampling and transported directly to the laboratory. Upon arrival, the soil was sieved through a 2 mm mesh to ensure uniformity. The mesh was sterilized with 75% ethanol after each sieving to prevent contamination. This process ensured that the samples were properly preserved and prepared for subsequent analysis. This sieved soil was then classified into two equal portions: one portion was air-dried for the purpose of conducting soil property measurements, while the other was freeze-dried and preserved at a temperature of −40°C, specifically reserved for future molecular analyses.

### 2.2 Climate data collections, and plant parameters and soil property measurements

The MAT and MAP data were sourced from the National Meteorological Information Center of China. The AI for each sampling site was computed following the formula proposed by Zorner et al. (2008). Field surveys recorded plant coverage and species richness, with all aboveground vegetation within each 1 m × 1 m quadrat being harvested, dried at 65°C, and subsequently weighed to determine aboveground biomass. For belowground biomass, root samples were gathered with a 9 cm diameter root corer to a depth of 10 cm, post-litter layer removal, and dried at 65°C.

Soil pH and conductivity measurements were performed on a soil-water suspension (ratio 1:2.5, w/v) utilizing an Ultrameter II 6PFCE instrument from MYRON, USA. Using a 2 M KCl solution in a ratio of 5:1 (solution:soil), NH_4_^+^-N and NO_3_^−^-N were extracted and subsequently explored on a continuous-flow analyzer (Skalar in Breda, Netherlands). Soil total carbon (TC) and nitrogen (TN) content was analyzed via an elemental analyzer (Multi-N/C 2100, Analytik Jena AG, Germany), while total phosphorus (TP) was quantified using the molybdenum blue method outlined by Murphy and Riley (1962). Total potassium (TK) concentrations were determined after digestion with HNO_3_ and HF, followed by quantification through ICP-OES (Optima 5,300 DV, Perkin Elmer, Waltham, MA, USA). Available P was assessed with the use of Olsen’s method (Olsen et al., 1954). Soil texture, comprising clay, silt, and sand fractions, was identified in accordance with the hydrometer method detailed by Bouyoucos (1962). Furthermore, soil TC was measured employing the K_2_Cr_2_O_7_-H_2_SO_4_ oxidation method as depicted by Nelson and Sommers (1996). The chloroform fumigation extraction method, as described by Brookes et al. (1985) and Zhu et al. (2021), was employed to determine the microbial biomass C and N in soil samples. Specifically, the simultaneous quantification of organic C and total N in 0.5 M K_2_SO_4_ extracts of both fumigated and un-fumigated samples was conducted using a TC/TN analyzer (Model Multi-N/C 2100, Analytik Jena AG, Germany). Following this, we calculated microbial biomass C and N, taking into account the differences in extractable organic C and total N between the two sets of samples, with a correction factor of 0.45 applied to account for unrecovered biomass, as per the recommendation by Jenkinson et al. (2004). Microbial biomass C, N and P were measured by the chloroform fumigation-extraction method (Brookes et al., 1982; Brookes et al., 1984; Brookes et al., 1985). To calculate the microbial biomass for each element, we determined the differences between the fumigated and unfumigated soil samples. Following the procedures outlined in the earlier research, we corrected for extraction efficiency by dividing the calculated microbial biomass C, N, and P by 0.45, 0.54, and 0.40, respectively (Brookes et al., 1982; Brookes et al., 1984; Brookes et al., 1985). The comprehensive data pertaining to these measurements are detailed in Supplementary Tables S2, S3.

### 2.3 DNA extraction, *pmoA* gene amplification, sequencing and bioinformatics analysis

The extraction of genomic DNA was performed from 0.25 grams of dried soil utilizing the PowerSoil DNA Isolation kit from QIAGEN, Germany. For the amplification of the *pmoA* gene, the primer pair A189f (5’-GGNGACTGGGACTTCTGG−3′) and A682r (5’-GAASGCNGAGAAGAASGC-3′) was employed, as depicted by Kou et al. (2021). The primer A189f was modified at its 5′ end with a unique 12 bp barcode to enable the differentiation of samples. The PCR procedure for each soil sample involved triplicate amplifications of the *pmoA* genes in a 25 μL reaction mixture, adhering to the protocol outlined in previous study by Li et al. (2024). This entailed an initial denaturation step at 94°C for 4 min, followed by 35 cycles which are consisted of denaturation at 94°C for 30 s, annealing at 55°C for 30 s, and extension at 72°C for 45 s. A final extension step at 72°C for 10 min completed the PCR process. Then, the triplicate amplification products from each sample were combined and purified with the AxyPrepTM DNA Gel Extraction Kit sourced from the USA. After purification, all products with distinct barcodes were pooled in equal molar ratios. Subsequently, these pooled samples underwent Illumina Miseq sequencing (2 × 300 bps), adhering strictly to the manufacturer’s guidelines.

Raw reads were explored with QIIME 2 (Bolyen et al., 2019). Within this pipeline, the DADA2 algorithm was deployed for denoising purposes (Callahan et al., 2019). Sequences that failed to meet the default quality standards, based on Phred scores, or were identified as chimeric were subsequently discarded. Resulted amplicon sequence variants (ASVs) underwent further scrutiny through frameshift checking, facilitated by FrameBot (Wang et al., 2013). This process led to the deduction of amino acid sequences, which were then used for taxonomic classification against a local database (Dumont et al., 2014), applying a BLASTP search with an e-value threshold of <1e^−5^ and a minimum percent identity of >85%. ASVs that exhibited similarity to AOB-like sequences, known for their phylogenetic proximity to *amoA* genes (Li et al., 2021; Lueke and Frenzel, 2011; Dumont et al., 2014), were filtered out from the dataset. Finally, to ensure uniformity across samples, the sequence count for each soil sample was rarefied to 800, with singletons removed prior to this step.

### 2.4 Statistical analysis

Statistical analyses were performed out in R software, version 4.2.2 (R Core Team, 2023). We employed two distinct matrices to quantify *β*-diversity based on an ASV table: the abundance-based Bray-Curtis distance matrix, generated with the ‘vegdist’ function from the ‘vegan’ package (Oksanen et al., 2023), and the *β* mean nearest taxon distance (*β*MNTD) matrix, calculated through the ‘comdistnt’ function in the ‘picante’ package (Kembel et al., 2010). These matrices, respectively, capture the taxonomic and phylogenetic dimensions of *β*-diversity. To investigate the distance-decay pattern, which relates *β*-diversity to spatial distance, we applied linear regression analysis across all samples. This comprehensive approach considers the entire range of microhabitats, thereby mitigating the influence of environmental filtering and approximating the true effects of spatial dispersal on the distance-decay relationship. For assessing variations in the slopes of these relationships among different microbial groups, we utilized the ‘lsmeans’ package (Lenth, 2016). Additionally, a single *β*-diversity value for each sample was derived using the ‘betadisper’ function within the ‘vegan’ package, providing a concise representation of diversity within the dataset.

To investigate the expected relationship between species pool (*γ* diversity) and *β*-diversity in the absence of specific assembly processes, we employed a random sampling simulation approach, as outlined by Kraft et al. (2011). Subsequently, we analyzed the observed *γ*-*β*-diversity relationship derived from sequencing data through linear regression methods. For quantifying the observed *γ*-diversity, we utilized the ‘specpool’ function within the ‘vegan’ package. As for *β*-diversity, we represented it by the betadispersion indices derived from both Bray-Curtis distance and *β*MNTD matrices, computed with the ‘betadisper’ function from the ‘vegan’ package. If the local species pool is a primary determinant of *β*-diversity, then the expected and observed *γ*-*β*-diversity relationships ought to exhibit a close correspondence.

To quantify the extent to which community assembly processes contribute to *β*-diversity patterns, this study employed null model analyses. Specifically, we calculated the *β* nearest taxonomic index (*β*NTI), which gauges the deviation of observed *β*MNTD from its expected value under null models. Additionally, we computed the Raup-Crick (RCbray) metric, a standardized assessment of Bray–Curtis dissimilarity, as outlined by Stegen et al. (2013), with the purpose of further evaluating community assembly mechanisms. Specifically, a *β*NTI value greater than +2 or less than −2 indicates that *β*-diversity is primarily driven by variable or homogeneous selection, respectively. Alternatively, when |*β*NTI| is less than 2 and the RCbray metric is less than −0.95, it suggests that homogenizing dispersal predominates in shaping *β*-diversity. Conversely, if |*β*NTI| remains below 2 but the RCbray metric exceeds +0.95, it implies that dispersal limitation is the dominant factor influencing *β*-diversity. Lastly, when |*β*NTI| and |Raup-Crick_bray_| both fall below 0.95, it signifies that ecological drift plays a primary role in determining *β*-diversity patterns, as per the framework proposed by Stegen et al. (2013).

To assess biotic interactions, we employed network analysis (Barberan et al., 2014; Ma et al., 2016), using the ‘igraph’ package (Csardi and Nepusz, 2006) for network construction. Our networks were based on relative abundance data derived from *pmoA* amplicon sequencing, with a threshold of 0.01% relative abundance applied to filter amplicon sequence variants (Xu et al., 2021). We then computed Spearman correlation coefficients across all possible pairs of these variants within the samples. Applying random matrix theory, we determined a correlation coefficient cut-off of 0.68, accompanied by a false discovery rate-adjusted *p*-value of less than 0.001. Subsequently, we calculated different network topological properties, including the number of nodes, edges, average degree, average path length, clustering coefficient and connectivity (Supplementary Table S3), which were considered potential proxies for quantifying biotic interactions.

To evaluate the intricate relationships between *β*-diversity and various community assembly processes (excluding biotic interactions), as well as those involving biotic interactions, we utilized multiple regression on distance matrices (MRM) analysis, as described by Lichstein (2007). For the community assembly process model (excluding biotic interactions), the *β*NTI matrices as explanatory variable. Conversely, the model assessing biotic interactions incorporated euclidean distances derived from network topological properties, specifically the number of nodes, edges, modules, average degree, path length, clustering coefficient, connectivity and modularity. The R^2^ value obtained from the MRM models served as an indicator of the proportion of *β*-diversity explained by the respective community assembly processes. Furthermore, these MRMs were extended to quantify the importance of factors modulating *pmoA β*-diversity.

## 3 Results

### 3.1 The *β*-diversity pattern of the *pmoA* community

**Figure 2:**
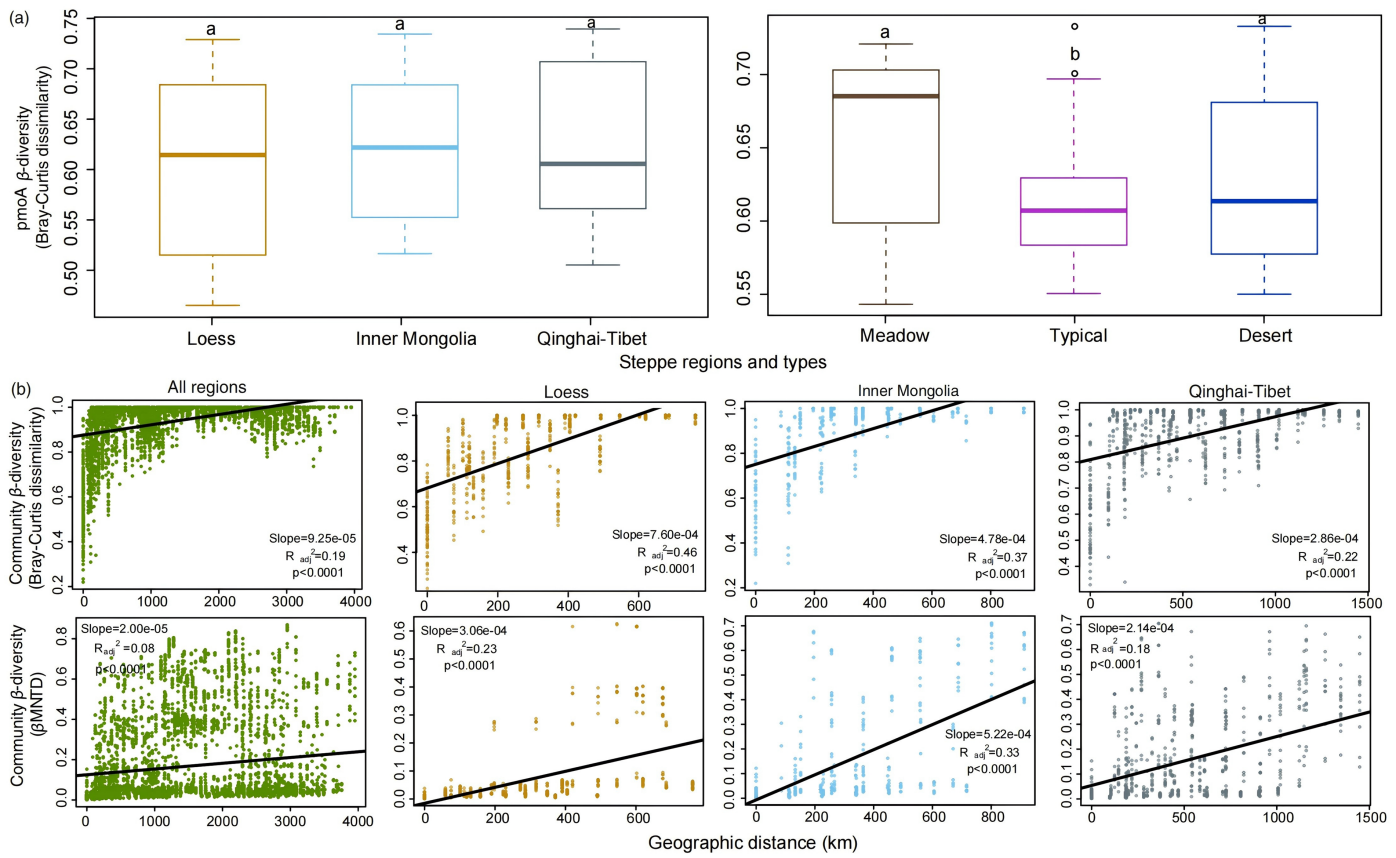
Variation patterns of *pmoA β*-diversity based on taxonomic Bray–Curtis dissimilarity between different steppe regions and types **(A)**. Geographical distance decay patterns of *pmoA* communities across all regions and within each individual region, based on Bray-Curtis distance and *β* mean nearest taxonomic distance (*β*MNTD) matrices **(B)**.

The analysis revealed no significant difference (*p* > 0.05) in *pmoA β*-diversity among the Loess, Inner Mongolia and Qinghai-Tibet regions, whereas a notable decrease in *pmoA β*-diversity was found in typical steppes compared to desert and meadow steppe types (Figure 2A). Moreover, an obvious positive correlation (*p* < 0.001) was identified between *pmoA β*-diversity and increasing spatial distances between samples (Figure 2B; Supplementary Figure S1). Nevertheless, the magnitude of this distance-decay relationship varied obviously among distinct steppe regions and types, with the Qinghai-Tibet region exhibiting a significantly flatter slope compared to Loess and Inner Mongolia (Figure 2B and Supplementary Figure S1, Table 1). Notably, when comparing all regions collectively, the overall slope was obviously flatter than that observed specifically in Loess, Inner Mongolia, and Qinghai-Tibet or any of the steppe types individually (Figure 2B and Supplementary Figure S1, Table 1). Additionally, except in the meadow steppe, the phylogenetic *β*-diversity exhibited weaker decay rates compared to the taxonomic *β*-diversity (Figure 2B; Supplementary Figure S1).

**Table 1:** Differences in slopes of distance-decay pattern regarding different regions and steppe types.

Distance-matrices	Comparisons	Slope differences^a^	Standard errors	*df*	P value^b^
Bray-curtis	LP vs. IMG	1.11E-04	2.59E-05	6,666	***
	LP vs. QTP	2.93E-04	1.89E-05	6,666	***
	LP vs. All	3.49E-04	1.68E-05	6,666	***
	IMG vs. QTP	1.82E-04	2.15E-05	6,666	***
	IMG vs. All	2.39E-04	1.98E-05	6,666	***
	QTP vs. All	5.70E-05	8.66E-06	6,666	***
	Desert vs. Meadow	8.85E-06	5.92E-06	6,979	ns
	Desert vs. All	4.74E-05	4.13E-06	6,979	***
	Desert vs. Typical	5.75E-06	5.26E-06	6,979	ns
	Meadow vs. All	3.85E-05	4.67E-06	6,979	***
	Meadow vs. Typical	-3.10E-06	5.69E-06	6,979	ns
	All vs. Typical	-4.16E-05	3.79E-06	6,979	***
*β*MNTD	LP vs. IMG	−2.23E-04	5.43E-05	6,994	***
	LP vs. QTP	8.93E-05	3.99E-05	6,994	ns
	LP vs. All	2.57E-04	3.53E-05	6,994	***
	IMG vs. QTP	3.13E-04	4.53E-05	6,994	***
	IMG vs. All	4.81E-04	4.14E-05	6,994	***
	QTP vs. All	1.68E-04	1.89E-05	6,994	***
	Desert vs. Meadow	−1.63E-04	1.10E-05	6,979	***
	Desert vs. All	−1.58E-05	7.68E-06	6,979	ns
	Desert vs. Typical	5.89E-06	9.78E-06	6,979	ns
	Meadow vs. All	1.47E-04	8.68E-06	6,979	***
	Meadow vs. Typical	1.69E-04	1.06E-05	6,979	***
	All vs. Typical	2.17E-05	7.05E-06	6,979	*

LP, Loess plateau; IMG, Inner Mongolian grassland; QTP, Qinghai-Tibetan plateau.

a Negative and positive values indicate smaller and greater slopes of the former than the latter, respectively.

b Significant codes are as follows: ns, non-significant, *p < 0.05, ***P < 0.001.

### 3.2 Relative importance of species pool, community assembly processes and biotic interactions in shaping *β*-diversity patterns

**Figure 3:**
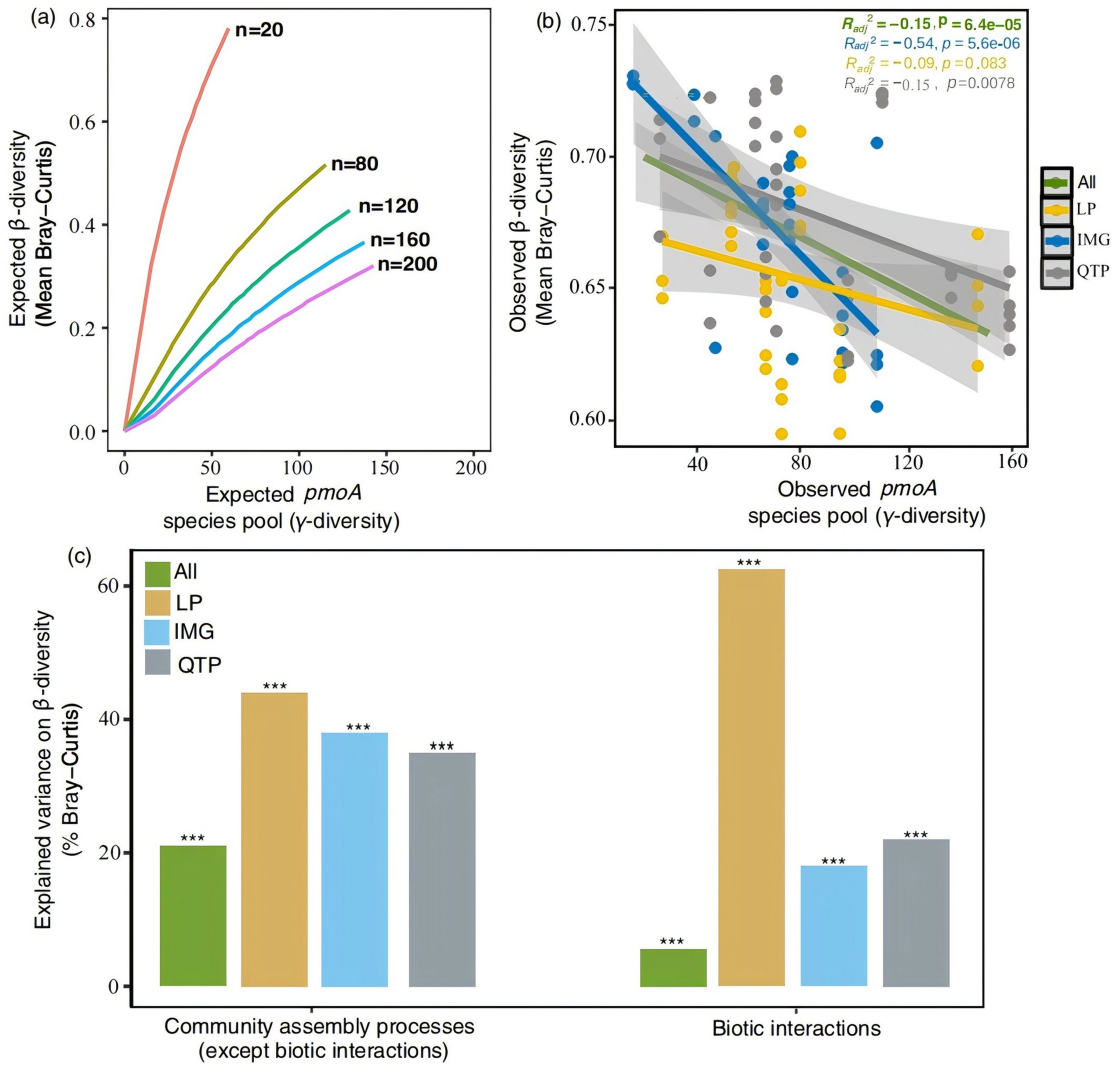
Relative contributions of species pool, community assembly processes (except biotic interactions), and biotic interactions in regulating *β*-diversity patterns. **(A)** Expected relationships between *β*-diversity and species pool (*γ*-diversity) when n individuals are randomly sampled from a fixed-sized species pool. **(B)** Observed relationships between *β*-diversity based on Bray-Curtis and *γ*-diversity. **(C)** Explained variability of community assembly processes (except biotic interactions) and biotic interactions, represented by co-occurrence network topological features, on *β*-diversity (based on Bray-Curtis distance). ***p < 0.001.

The observed negative or unrelated *γ*–*β*-diversity relationship, persistent across different steppe regions and types (Figures 3A,B; Supplementary Figure S2A), indicates that the species pool does not significantly influence *β*-diversity. However, community assembly processes, excluding biotic interactions, contributed varying degrees to *pmoA β*-diversity variations: 44.2% in Loess, 38.5% in Inner Mongolia, 35.1% in Qinghai-Tibet, and 21.2% when considering all regions combined (Figure 3C). Similarly, for meadow, typical, and desert steppes, these processes explained 48.2, 4.3, and 9.3% of *pmoA β*-diversity variations, respectively (Supplementary Figure S2B). Additionally, biotic interactions among *pmoA* species played a substantial role in *β*-diversity variations, accounting for 62.3% in Loess, 18.3% in Inner Mongolia, 21.5% in Qinghai-Tibet, and 5.6% across all regions combined (Figure 3C). In the context of steppe types, these interactions explained 10.1% of *β*-diversity variations in meadow steppes, 22.4% in typical steppes, and 11.8% in desert steppes (Supplementary Figure S2B).

### 3.3 Community assembly processes governing *pmoA β*-diversity

**Figure 4:**
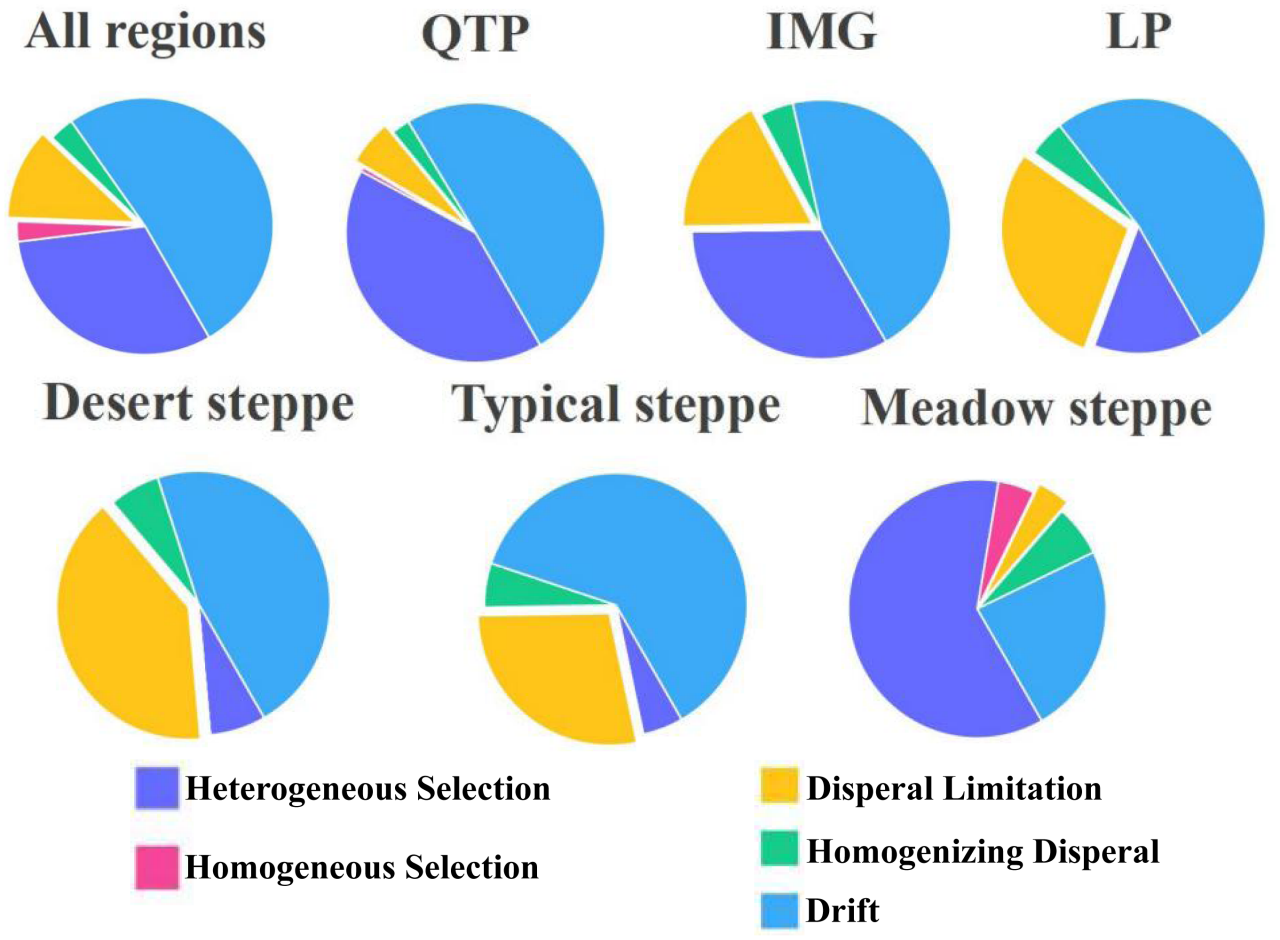
The contributions of community assembly processes shaping the methanotrophic community.

Our results revealed that stochastic processes, notably drift and dispersal limitation, contributed over 58.3% in all cases examined (Figure 4). Specifically, drift emerged as the dominant process in all scenarios except for meadow steppes, where heterogeneous selection played a more pivotal role. Apart from drift, both dispersal limitation and heterogeneous selection contributed to explaining *pmoA β*-diversity variations across all cases examined, albeit with varying degrees of relative importance (Figure 4). In terms of regional differences, dispersal limitation exhibited a higher contribution in the LP transect, while its influence was lesser in the QTP region (Figure 4). Conversely, heterogeneous selection displayed an opposite trend, being less pronounced in the LP transect and more significant in the QTP (Figure 4). Regarding steppe types, stochastic processes, including dispersal limitation, contributed the most in desert steppes, followed by typical steppes, and least in meadow steppes (Figure 4). Similarly, heterogeneous selection followed an inverse pattern, with the highest contribution in meadow steppes and lower contributions in desert and typical steppes (Figure 4).

### 3.4 Key environmental factors shaping *pmoA β*-diversity

Key factors shaping this functional community appeared to vary by scale and region. At larger spatial scales (e.g., all regions together or each of the steppe type), MAT was the dominant factor (Table 2). However, within individual regions, soil pH was most influential in Inner Mongolia and Qinghai-Tibet, while MAP was key in the Loess region (Table 2). Furthermore, these key environment factors showed the strongest correlation with *β*NTI and community assembly process (Supplementary Figure S3).

**Table 2:** Multiple regressions on distance matrices (MRM) were employed to estimate the relatively explained variance of environmental factors on methanotrophic community structure (*β*-diversity).

Steppe regions or types	Variable	R^2^	*p*
All regions	MAT	0.179	0.0001
	pH	0.161	0.0001
	AI	0.161	0.0001
Loess	MAP	0.735	0.0001
	AI	0.727	0.0001
	PC	0.474	0.0001
Inner Mongolia	pH	0.326	0.0001
	AI	0.282	0.0001
	MAP	0.254	0.0001
Qinghai-Tibet	pH	0.496	0.0001
	NH_4_^+^-N	0.469	0.0001
	TOC	0.453	0.0001
Meadow	pH	0.561	0.0001
	MAT	0.524	0.0001
	TOC	0.418	0.0001
Typical	MAT	0.558	0.0001
	PC	0.371	0.0001
	MBC	0.245	0.0001
Desert	MAT	0.534	0.0001
	AI	0.358	0.0001
	TP	0.197	0.0001

MAT, Mean annual temperature; AI, Aridity index; MAP, Mean annual precipitation; PC, Plant coverage; TOC, Total organic carbon; MBC, Microbial biomass carbon; TP, Total phosphorus. In the table, for each region and grassland type, only the top three factors with the highest explanatory power, sorted in descending order of their explanatory degrees, are displayed.

## 4 Discussion

### 4.1 Scale-dependent patterns of *pmoA β*-diversity

The obtained findings provide compelling evidence for the distance-decay pattern in functional *pmoA* communities across various regions and habitats. The distinct variations in *pmoA β*-diversity with the increase of geographical distances parallel trends observed in macro-organisms and other microbial communities, such as total soil bacteria, fungi, and functional *phoD* communities (Averill et al., 2019; Ferenc et al., 2014; Morlon et al., 2008; Wang et al., 2017; Xu et al., 2021). Notably, the slope of this relationship varies with spatial scale, with longer distances between sampling sites in the Loess, Inner Mongolia, and Qinghai-Tibet regions contributing to flatter slopes. Moreover, when considering all regions together, the slope flattens, suggesting a more gradual increase in *β*-diversity over larger distances. This finding aligns with previous studies on *phoD* and fungi (Averill et al., 2019; Xu et al., 2021), reinforcing the idea that larger spatial scales tend to elicit smaller changes in *β*-diversity. Furthermore, meadow steppes exhibit a flatter slope compared to typical and desert steppes, underlining habitat-specific variations in microbial distance-decay patterns.

Furthermore, the weaker decay rates observed in phylogenetic *β*-diversity compared to taxonomic *β*-diversity (except in the meadow steppe), align with previous findings reported by Xu et al. (2024). This can be attributed to phylogenetic niche conservatism during speciation events spanning macroevolutionary timescales (Crisp and Cook, 2012). However, in the meadow steppe, we observed higher decay rates in phylogenetic *β*-diversity relative to taxonomic *β*-diversity. These discrepant patterns are likely due to diverse community assembly mechanisms, arising from the intricate interactions among various microhabitats, the unique environmental characteristics across geographical locations, and the environmental preferences exhibited by specific microbial taxa (Logue et al., 2015; Nguyen et al., 2021).

### 4.2 Community assembly processes, but not species pool, determine spatial patterns of *pmoA β*-diversity

Theoretically, habitats with larger species pools need to offer a wider array of candidate species for colonization, leading to greater *β*-diversity among samples (Kraft et al., 2011). Nevertheless, the obtained findings indicated that the size of the *pmoA* species pool was not a crucial factor in explaining variations in *β*-diversity. This observation aligns with similar findings in *phoD* communities within the same region (Xu et al., 2024), potentially attributable to the predominant influence of community assembly processes on *pmoA* communities. Previous studies also reported that the relative significance of the species pool to *β*-diversity could be modulated by the intensity of community assembly processes, including dispersal, environmental filtering, and biotic interactions (Chase and Myers, 2011; Cornell and Harrison, 2014; Stegen et al., 2012, 2013, 2015). When these processes attain a certain level of strength, the influence of the species pool may diminish in significance. Indeed, our study underscores the pivotal role of community assembly mechanisms (both deterministic and stochastic) and biotic interactions in explaining *β*-diversity patterns in *pmoA* communities. However, a previous study conducted in desert steppe suggested that species pools of bacterial and diazotrophic communities were crucial for diazotrophic *β*-diversity (Wang et al., 2021; Xu et al., 2024). Consequently, the correlation between species pool and *β*-diversity appears to be contingent upon organism type, habitat type, and the spatial scales considered.

When compared with the species pool, both deterministic and stochastic processes exhibited obvious roles in shaping the *pmoA* community assembly across diverse scales. This suggests that niche differentiation, driven by both historical factors (e.g., geographical isolation spanning extensive historical timescales) and contemporary factors (e.g., environmental filtering), is probably paramount in identifying the spatial patterns of *pmoA β*-diversity across the steppes of China. However, the relative contributions of stochastic processes (drift and dispersal limitation) and deterministic processes (heterogeneous selection) vary with scale and grassland type. Our study presents a novel perspective, first elucidating that ecological drift, rather than the conventionally assumed dispersal limitation and heterogeneous selection at larger scales (Kou et al., 2021; Peay et al., 2010; Zhang et al., 2024), predominantly governs the explanation of *pmoA β*-diversity across diverse scales and grassland types, with exception of meadow steppes. This finding stems from the understanding that ecological drift assumes greater significance in shaping microbial community structures within environments characterized by weak environmental selection, which often correlate with low microbial richness and abundance (Ramoneda et al., 2020). In arid and semi-arid habitats specifically, soil aMOBs, already challenged by their limited richness and abundance, are highly susceptible to drift, as even minor reductions in their numbers can lead to extinction (Chase, 2007). Furthermore, the presence of vegetation patches in these habitats, particularly in desert steppes, amplifies the role of ecological drift in shaping the methanotrophic community, as the reduced number of individuals within these patches heightens the risk of local species extinction and intensifies the impact of drift on community dynamics (Lanta et al., 2018; Vellend and Agrawal, 2010). When a species’ population dwindles, it becomes increasingly vulnerable to environmental fluctuations and stochastic events, due to the smaller gene pool and reduced ecological interactions among the remaining individuals (Gilbert and Levine, 2017). Thus, our research underscores the preeminence of drift as the primary process underpinning the formation of *pmoA β*-diversity in steppes spanning steppe of China. Thus, our research highlights the overriding significance of drift as the principal process underlying the establishment of *pmoA β*-diversity across the steppes of China. In contrast, for meadow steppes, deterministic processes predominate in the assembly of *pmoA* communities, aligning with previous studies conducted in the QTP (Kou et al., 2020). Consequently, we propose that the diversity of habitat types arising from variations in environmental conditions, including those associated with arid and semi-arid environments, may lead to the formation of *pmoA β*-diversity through distinct community assembly processes.

In addition to drift, dispersal limitation and heterogeneous selection all contribute to varying degrees in explaining *pmoA β*-diversity across different scales and grassland types, albeit with differing relative importances. For instance, on the QTP, heterogeneous selection accounts for the largest proportion compared to other scales, with soil pH emerging as an important factor. This aligns with previous studies in the QTP grassland soils, which identified soil pH as a key determinant influencing *pmoA* community assembly (Li et al., 2021, 2024). Indeed, our results also demonstrate a significant correlation between *β*NTI and pH, indicating that changes in pH significantly alter the phylogenetic structure of *pmoA β*-diversity through heterogeneous selection. Conversely, in LP, heterogeneous selection is minimal while dispersal limitation is maximal compared to other scales. Moreover, we identified MAP as a pivotal factor governing community assembly, consistent with prior research that MAP controls bacterial community assembly (Yang et al., 2022). MAP exerts its influence on microbial community assembly through several mechanisms. Firstly, the vast precipitation differences across LP locations modify habitat conditions, subsequently altering microbial community *β*-diversity, as higher precipitation levels enhance soil moisture. Furthermore, different textured soils in LP are capable of retaining varying degrees of moisture (such as coarse-textured soil in desert steppe tend to retain less water compared to finer-textured soils) (Schimel, 2018). These characteristics may enhance the effect of dispersal limitation. Consequently, microbial communities, especially aMOBs, which thrive in conditions of optimal water availability that not only cater to their physiological activity but also facilitate the exchange of oxygen and CH_4_ within the soil, are notably more vulnerable to limitations imposed by their local environments. Our results further support this notion, revealing that variations in MAP significantly impact the phylogenetic structure of *pmoA β*-diversity and community assembly through dispersal limitation. Regarding grassland types, dispersal limitation plays a stronger role in desert grasslands, aligning with previous findings on soil fungi and *phoD*-harbouring communities in desert biocrust systems, which suggested that microbial communities in these systems likely exhibit low dispersal rates (Stegen et al., 2012, 2013, 2015). Furthermore, this dominance of dispersal limitation can be attributed to the low environmental heterogeneity in deserts, characterized by coarse-textured soil, low water availability, and relatively uniform vegetation (Allen et al., 2020). Despite the weak heterogeneous selection in desert steppes, MAT emerges as the most critical factor influencing *pmoA β*-diversity. Temperature fluctuations may affect micro-environmental conditions such as soil moisture and pH in desert regions, thereby influencing community *β*-diversity. Indeed, our results indicate that increased MAT differences significantly enhance *pmoA β*-turnover, suggesting that MAT primarily impacts the local assembly processes of *pmoA* communities in desert areas. However, across similar spatial scales, the contribution of dispersal limitation to *pmoA* community assembly diminishes from desert to typical and then to meadow steppes, indicative of an increasing habitat effect on dispersal limitation. Additionally, a lower contribution from stochastic dispersal limitation may occur in habitats with stronger environmental filtering, as exemplified in the meadow steppe. We further reveal that soil pH is the most important factor which can shape the *β*-diversity patterns of *pmoA* communities in meadow steppes, consistent with previous research in these ecosystems. Notably, significant changes in soil pH can significantly alter the phylogenetic turnover of *pmoA β*-diversity in meadow steppes. Thus, our study underscores the predominance of community assembly processes in regulating *pmoA* community *β*-diversity across China’s grasslands, albeit with varying contributions from stochastic processes (drift and dispersal limitation) and deterministic processes (heterogeneous selection) depending on scale and grassland type. The obtained findings can improve our understanding of the mechanisms underlying the geographical distributions of soil methanotrophs at broad scales.

## 5 Conclusion

This study has provided new insights into the community assembly mechanisms of *pmoA* communities, revealing the presence of distance-decay patterns in their *β*-diversity. Notably, our findings underscore the dominance of local community assembly mechanisms over regional species pools in shaping soil *pmoA β*-diversity patterns across the steppes of China. However, the relative significance of these assembly processes, encompassing dispersal limitation, drift, environmental filtering, and biotic interactions, varies considerably based on spatial scales and steppe types. Moreover, fluctuations in environmental conditions serve as mediators, influencing the intensity of distinct community assembly processes, thereby driving *β*-diversity variation. Collectively, our research offers direct evidence emphasizing the pivotal role of local community assembly processes in delineating the geographical patterns of soil *pmoA β*-diversity along precipitation-temperature gradients within the Northern Hemisphere’s steppe ecosystems. These insights hold profound implications for advancing our understanding of microbial ecology and inform strategies for the conservation and management of these vital ecosystems.

## Data Availability

The datasets presented in this study can be found in online repositories. The names of the repository/repositories and accession number(s) can be found in the article/Supplementary material.
