# Supplementary material for: Assembly mechanisms, not species pool, shape β-diversity of soil methanotrophic communities in steppes of China
Source: Front Microbiol. 2025 Jan 20;15:1522319. doi: 10.3389/fmicb.2024.1522319 (PMC11788413; doi:10.3389/fmicb.2024.1522319)
Supplement: Supplementary file 1 [file Data_Sheet_1.DOCX]

**Assembly mechanisms, not** **species pool, shape *β*-diversity of** **soil methanotrophic communities in steppes of China**


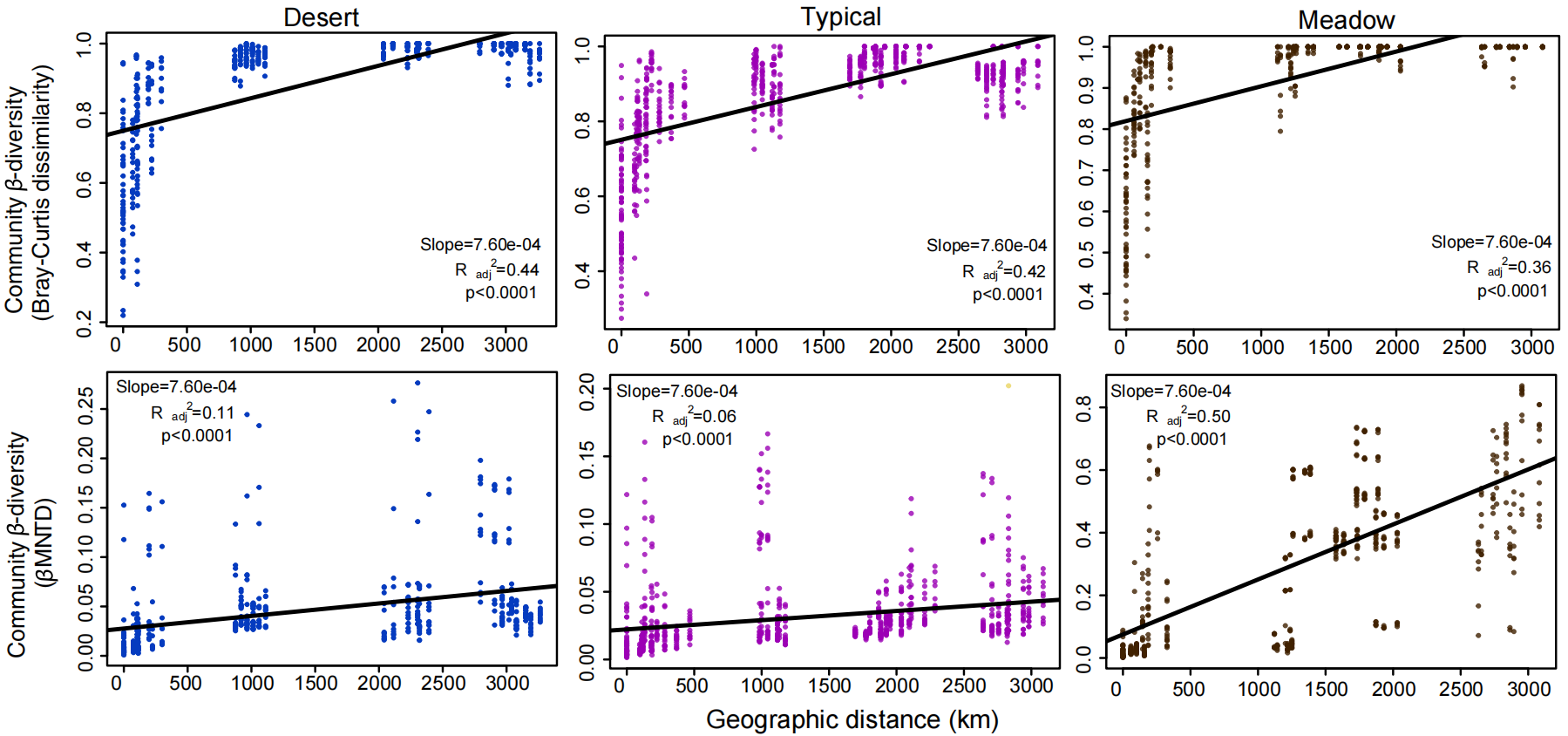


Figure S1 Geographical distance decay patterns of *pmoA* communities across all regions and each individual region, based on Bray-Curtis distance and *β* mean nearest taxonomic distance (*β*MNTD) matrices (b).


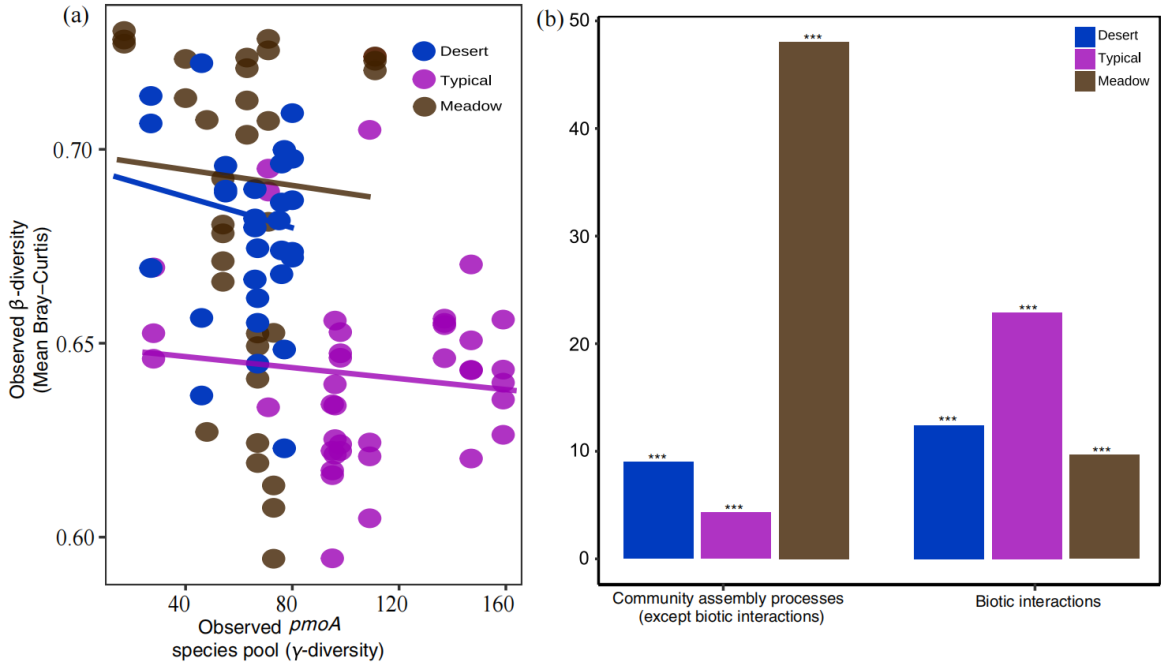


Figure S2 (a) Observed relationships between *β*-diversity based on Bray-Curtis and *γ*-diversity. (b) Explained variability of community assembly processes (except biotic interactions) and biotic interactions, represented by co-occurrence network topological features, on *β*-diversity (based on Bray-Curtis distance). ****p*< 0.001.


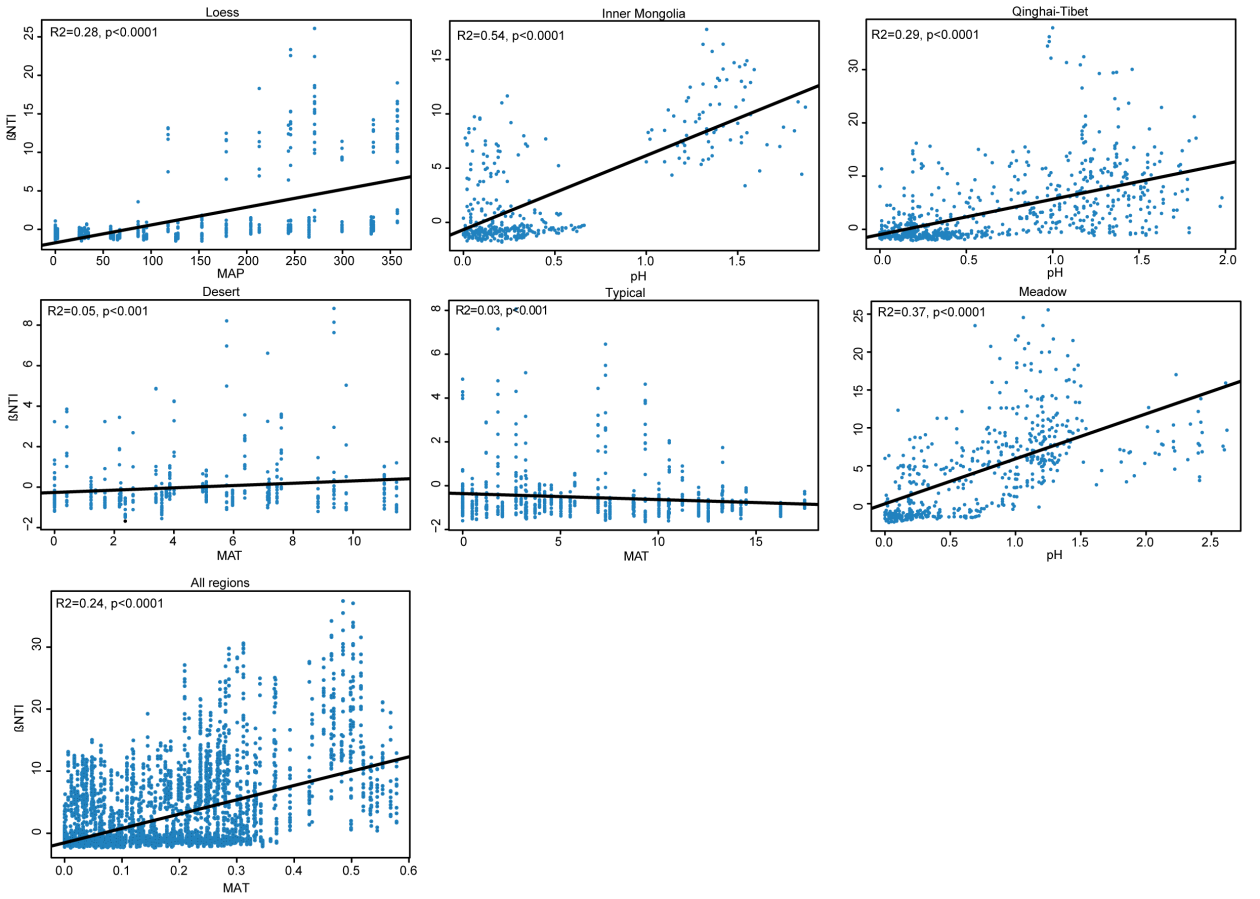


Figure S3 Relationships between *β*NTI and key environment factors across all regions and each individual region. R-squared and *p*-values are calculated based on the quadratic polynomial fits. *β*NTI represent *β*–nearest taxon index.

Table S1 Information of sampling sites, soil and steppe types, dominant plant species, geographic locations and climate parameters across Loess plateau (LP), Inner Mongolian (IMG) and Qinghai- Tibet Plateau (QTP) transects.

| Plots ID | Region | Longitude | Latitude | MAT (°C) | MAP (mm) | AI | Soil types | Steppe types | Dominant plant species |
| --- | --- | --- | --- | --- | --- | --- | --- | --- | --- |
| LP1 | Loess | 113.36° | 36.29° | 11.85 | 563.88 | 0.43 | Cinnamonic soils | Temperate meadow | *L. chinensis, Bothriochloa ischaemum, Lespedeza bicolor*、Carex spp. |
| LP2 | Loess | 112.29° | 35.99° | 9.96 | 591.18 | 0.4 | Cinnamonic soils | Temperate meadow | *Carex* spp., Themeda japonica, Artemisia gmelinii |
| LP3 | Loess | 111.64° | 35.99° | 10.66 | 566.12 | 0.36 | Cinnamonic soils | Temperate meadow | *Poa annua, Artemisia argyi, L. bicolor* |
| LP4 | Loess | 110.18° | 36.07° | 10.72 | 533.36 | 0.37 | Dark loessial soils | Temperate typical steppes | *A. gmelinii, B. ischaemum, Wikstroemia chamaedaphne* |
| LP5 | Loess | 109.24° | 36.74° | 9.5 | 498.89 | 0.35 | Dark loessial soils | Temperate typical steppes | *Lespedeza daurica, B. ischaemum, A. gmelinii* |
| LP6 | Loess | 107.92° | 36.93° | 7.46 | 438.09 | 0.32 | Dark loessial soils | Temperate typical steppes | *Cleistogenes* spp., A. gmelinii, S. capillata |
| LP7 | Loess | 107.18° | 37.58° | 5.22 | 395.14 | 0.28 | Dark loessial soils | Temperate typical steppes | *L. chinensis, Carex* spp., Artemisia scoparia |
| LP8 | Loess | 105.78° | 37.42° | 5.87 | 320.35 | 0.15 | Sierozems | Temperate desert steppes | *Cleistogenes* spp., Reaumuria songarica, Alopecurus aequalis |
| LP9 | Loess | 104.92° | 37.44° | 7.56 | 233.88 | 0.13 | Sierozems | Temperate desert steppes | *Heteropappus altaicus, Cleistogenes* spp. |
| LP10 | Loess | 104.44° | 37.46° | 7.71 | 215.5 | 0.11 | Sierozems | Temperate desert steppes | *R. songarica, Suaeda glauca, E. Pilosa, Kalidium foliatum* |
| IMG1 | Inner Mongolia | 123.51° | 44.59° | 5.1 | 448.47 | 0.32 | Chernozems | Temperate meadow | *Chloris virgata, Leymus chinensis, Phragmites communis* |
| IMG2 | Inner Mongolia | 121.04° | 44.52° | 5.8 | 384.39 | 0.27 | Chernozems | Temperate meadow | *Setaria viridis, Ephedra sinica, Agropyron cristatum* |
| IMG3 | Inner Mongolia | 120.33° | 45.11° | 3.72 | 384.47 | 0.29 | Chernozems | Temperate meadow | *S. viridis, Cleistogenes* spp., L. chinensis |
| IMG4 | Inner Mongolia | 118.36° | 44.77° | 0.56 | 403.58 | 0.31 | Chestnut soils | Temperate typical steppess | *Stipa capillata, A. cristatum, L. chinensis, Carex* spp. |
| IMG5 | Inner Mongolia | 116.52° | 44.26 | 1.17 | 315.49 | 0.17 | Chestnut soils | Temperate typical steppess | *Chenopodium glaucum, S*. *capillata, L. chinensis, Salsola collina* |
| IMG6 | Inner Mongolia | 116.67° | 43.55° | 0.16 | 385.81 | 0.27 | Chestnut soils | Temperate typical steppess | *S*. capillata, Achnatherum sibiricum, L. chinensis |
| IMG7 | Inner Mongolia | 117.68° | 44.51° | 1.96 | 344.68 | 0.27 | Chestnut soils | Temperate typical steppes | *Carex* spp., Cleistogene spp., Anemarrhena asphodeloides, L. chinensis |
| IMG8 | Inner Mongolia | 114.89° | 44.01° | 0.1 | 281.18 | 0.17 | Brown pedocals | Temperate desert steppes | *S. collina, Allium polyrhizum, L. chinensis* |
| IMG9 | Inner Mongolia | 113.5° | 43.84° | 2.47 | 213.16 | 0.14 | Brown pedocals | Temperate desert steppes | *Allium mongolicum, Carex* spp., Tribulus terrestris, Eragrostis pilosa |
| IMG10 | Inner Mongolia | 112.15° | 43.63° | 3.69 | 168.29 | 0.12 | Brown pedocals | Temperate desert steppes | *T. terrestris, A. mongolicum, Corispermum hyssopifolium, S. collina* |
| QTP1 | Qinghai-Tibet | 95.45° | 31.46° | 0.41 | 619.86 | 0.6 | Alpine soils | Alpine meadow | *Carex* spp., Kobresia pygmaea, Potentilla saundersiana |
| QTP2 | Qinghai-Tibet | 93.53° | 31.85° | -1.5 | 536.77 | 0.63 | Alpine soils | Alpine meadow | *Polygonum viviparum, K. pygmaea, Carex* spp. |
| QTP3 | Qinghai-Tibet | 92.01° | 31.64° | -4.37 | 501.38 | 0.39 | Alpine soils | Alpine meadow | *K. pygmaea, P. saundersiana, Saussurea japonica* |
| QTP4 | Qinghai-Tibet | 90.74° | 31.38° | -6.76 | 526.89 | 0.35 | Alpine soils | Alpine typical steppes | *S*. capillata, Carex spp., Astragalus adsurgens |
| QTP5 | Qinghai-Tibet | 89.72° | 31.54° | -3.06 | 443.79 | 0.33 | Alpine soils | Alpine typical steppes | *S*. capillata, Carex spp., Leontopodium leontopodioides, Potentilla bifurca |
| QTP6 | Qinghai-Tibet | 87.82° | 31.87° | -2.57 | 389.09 | 0.21 | Alpine soils | Alpine typical steppes | *S*. capillata, Carex spp., Stipa purpurea |
| QTP7 | Qinghai-Tibet | 85.84° | 31.92° | -3.77 | 376.17 | 0.1 | Alpine soils | Alpine typical steppes | *L. leontopodioides, Carex* spp., S. purpurea |
| QTP8 | Qinghai-Tibet | 83.34° | 32.41° | -3.9 | 317.13 | 0.06 | Alpine soils | Alpine desert steppes | *P. annua, E. Pilosa, Astragalus propinquus, S*. capillata |
| QTP9 | Qinghai-Tibet | 81.23° | 32.30° | -3.49 | 291.42 | 0.08 | Alpine soils | Alpine desert steppes | *S*. capillata, A. propinquus, Carex spp., Artemisia desertorum |
| QTP10 | Qinghai-Tibet | 80.15° | 32.48° | -1.27 | 191.71 | 0.07 | Alpine soils | Alpine desert steppes | *Stipa tianschanica, S*. capillata, Ajania fruticulosa |

Table S2 Information of plant and soil properties across Loess plateau (LP), Inner Mongolian (IMG) and Qinghai- Tibet Plateau (QTP) transects.

| Plots ID | PC | AGB | RB | PLSH | pH | CON | BD | Clay | Silt | Sand |
| --- | --- | --- | --- | --- | --- | --- | --- | --- | --- | --- |
| LP1 | 86.25±4.79 | 116.35±27.4 | 0.35±0.11 | 1.06±0.28 | 8.08±0.03 | 233.80±19.81 | 1.39±0.08 | 11.40±0.76 | 44.38±2.05 | 44.21±1.43 |
| LP2 | 85.00±7.91 | 86.32±35.62 | 0.36±0.29 | 1.83±0.16 | 7.96±0.07 | 183.00±10.07 | 1.27±0.21 | 9.23±0.91 | 58.74±1.60 | 32.03±2.30 |
| LP3 | 90.00±5.00 | 201.32±81.75 | 0.57±0.24 | 1.35±0.56 | 8.10±0.06 | 246.86±22.04 | 1.13±0.06 | 10.58±0.31 | 43.07±0.71 | 46.34±0.41 |
| LP4 | 81.67±12.58 | 160.08±25.34 | 0.60±0.37 | 1.27±0.20 | 8.02±0.02 | 220.90±7.01 | 1.33±0.12 | 9.01±0.66 | 36.02±2.83 | 54.97±3.48 |
| LP5 | 84.00±6.52 | 118.41±26.39 | 0.57±0.31 | 1.77±0.20 | 8.16±0.05 | 208.28±33.15 | 1.25±0.05 | 4.39±0.80 | 17.65±3.03 | 77.95±3.81 |
| LP6 | 58.00±15.65 | 95.18±34.36 | 0.27±0.15 | 1.79±0.27 | 8.11±0.03 | 177.64±13.26 | 1.25±0.10 | 6.20±0.23 | 23.23±0.42 | 70.57±0.40 |
| LP7 | 53±2.74 | 50.22±11.47 | 0.35±0.26 | 1.50±0.42 | 8.11±0.13 | 287.2±109.33 | 1.46±0.04 | 4.07±0.26 | 15.96±1.50 | 79.77±1.77 |
| LP8 | 36.25±13.15 | 61.36±5.70 | 0.70±0.59 | 0.96±0.37 | 8.17±0.05 | 310.70±23.10 | 1.28±0.05 | 7.72±1.20 | 27.74±4.93 | 64.54±6.10 |
| LP9 | 23.80±12.68 | 78.15±89.54 | 0.38±0.50 | 1.72±0.23 | 7.91±1.36 | 132.56±5.43 | 1.41±0.05 | 2.75±1.04 | 9.92±4.09 | 87.33±5.11 |
| LP10 | 37.40±16.09 | 115.62±24.19 | 0.68±0.91 | 1.24±0.30 | 8.49±0.07 | 216.20±35.78 | 1.44±0.03 | 8.67±0.83 | 40.72±3.32 | 50.60±4.11 |
| IMG1 | 78.00±14.73 | 132.49±38.53 | 0.24±0.13 | 1.38±0.44 | 9.19±0.12 | 1100.37±249.68 | 1.75±0.17 | 4.21±1.18 | 31.23±5.33 | 64.56±6.51 |
| IMG2 | 68.00±10.00 | 101.18±23.32 | 0.47±0.07 | 2.01±0.13 | 7.57±0.17 | 132.10±2.30 | 1.94±0.07 | 3.22±0.15 | 22.37±0.24 | 74.41±0.39 |
| IMG3 | 82.50±8.50 | 131.26±27.53 | 0.42±0.17 | 1.36±0.02 | 7.65±0.21 | 264.78±170.13 | 1.28±0.04 | 6.85±0.73 | 51.60±1.63 | 41.54±2.36 |
| IMG4 | 64.20±12.15 | 70.53±24.49 | 0.53±0.21 | 1.12±0.27 | 7.45±0.17 | 165.36±66.28 | 1.73±0.17 | 5.31±0.36 | 29.79±1.38 | 64.90±1.56 |
| IMG5 | 15.40±6.02 | 28.14±10 | 0.16±0.10 | 1.41±0.22 | 7.44±0.32 | 127.20±28.21 | 1.03±0.09 | 6.58±0.92 | 30.77±3.41 | 62.65±3.27 |
| IMG6 | 41.20±5.26 | 65.46±13.42 | 0.35±0.18 | 1.81±0.21 | 7.79±0.09 | 223.24±58.35 | 1.31±0.13 | 6.82±0.97 | 34.24±2.88 | 58.94±3.49 |
| IMG7 | 48.75±10.21 | 42.18±10.62 | 0.58±0.43 | 1.55±0.09 | 7.77±0.19 | 273.73±66.55 | 1.40±0.09 | 6.11±1.63 | 40.45±4.52 | 53.44±3.20 |
| IMG8 | 17.33±8.74 | 39.09±9.64 | 0.54±0.39 | 1.48±0.25 | 7.93±0.04 | 409.33±97.98 | 1.56±0.16 | 5.53±0.79 | 33.52±4.62 | 60.96±5.41 |
| IMG9 | 24.14±28.49 | 16.94±4.08 | 0.09±0.03 | 0.94±0.18 | 7.91±0.19 | 239.98±82.91 | 1.68±0.14 | 4.39±0.67 | 27.90±2.31 | 67.71±2.68 |
| IMG10 | 11.25±0.96 | 13.71±0.27 | 0.18±0.10 | 1.40±0.10 | 7.91±0.10 | 146.48±23.25 | 2.15±0.29 | 2.25±0.62 | 12.30±2.98 | 85.44±3.59 |
| QTP1 | 75.00±17.80 | 115.2±51.42 | 3.20±5.03 | 2.34±0.25 | 7.28±0.41 | 399.05±135.21 | 1.28±0.19 | 13.83±0.88 | 54.83±3.08 | 31.34±3.16 |
| QTP2 | 86.00±12.14 | 124.72±3.86 | 6.91±10.7 | 1.39±0.36 | 6.80±0.18 | 180.25±111.93 | 1.05±0.08 | 9.29±0.87 | 39.43±1.69 | 51.28±2.25 |
| QTP3 | 72.50±6.45 | 42.59±17.10 | 2.03±0.43 | 1.15±0.17 | 6.94±0.15 | 80.44±19.00 | 1.21±0.11 | 5.20±3.51 | 23.67±15.84 | 71.13±19.29 |
| QTP4 | 30.00±9.13 | 21.16±8.28 | 0.58±0.29 | 1.53±0.15 | 8.02±0.01 | 216.70±6.16 | 1.46±0.21 | 5.03±1.05 | 15.87±3.42 | 79.11±4.46 |
| QTP5 | 29.00±5.48 | 70.24±44.68 | 0.22±0.12 | 1.5±0.19 | 8.05±0.03 | 157.14±6.44 | 1.40±0.14 | 11.39±1.20 | 31.27±7.28 | 57.34±8.27 |
| QTP6 | 26.60±2.07 | 41.52±19.01 | 0.31±0.29 | 1.27±0.33 | 8.17±0.03 | 186.42±11.71 | 1.60±0.12 | 5.25±1.15 | 12.20±1.64 | 82.55±2.73 |
| QTP7 | 29.67±2.52 | 72.53±16.35 | 0.49±0.18 | 1.37±0.10 | 8.46±0.15 | 1522.83±502.14 | 1.59±0.08 | 10.21±1.67 | 38.67±8.07 | 51.13±9.64 |
| QTP8 | 29.00±6.56 | 33.43±8.96 | 0.26±0.22 | 1.22±0.13 | 8±0.24 | 663.53±506.84 | 1.25±0.03 | 15.64±2.93 | 31.91±6.08 | 52.45±8.99 |
| QTP9 | 6.25±1.50 | 28.44±18.97 | 0.39±0.34 | 1.05±0.22 | 8.43±0.04 | 110.34±9.92 | 1.55±0.06 | 7.47±1.56 | 17.08±4.50 | 75.46±6.03 |
| QTP10 | 12.33±2.52 | 15.83±7.68 | 1.02±0.51 | 0.25±0.16 | 8.07±0.33 | 599±817.53 | 1.73±0.10 | 6.87±0.95 | 16.46±3.95 | 76.66±4.89 |

Table S2 Continued

| Plots ID | MBC | MBN | MBP | TOC | TC | TN | NH_4_^+^-N | NO_3_^-^-N | TP | TK | AP |
| --- | --- | --- | --- | --- | --- | --- | --- | --- | --- | --- | --- |
| LP1 | 352.31±56.77 | 53.14±6.98 | 8.03±3.40 | 1.23±0.11 | 2.32±0.12 | 0.12±0.01 | 1.52±0.13 | 25.95±5.39 | 0.65±0.04 | 18.05±0.42 | 3.76±1.11 |
| LP2 | 336.85±72.2 | 21.14±8.77 | 6.01±2.30 | 1.91±0.24 | 2.02±0.38 | 0.15±0.02 | 2.22±0.61 | 0.73±0.24 | 0.40±0.03 | 17.31±1.76 | 3.04±0.76 |
| LP3 | 191.11±59.76 | 18.71±5.18 | 5.53±1.91 | 1.82±0.24 | 3.26±0.35 | 0.15±0.02 | 2.75±0.56 | 3.84±2.54 | 0.52±0.05 | 16.88±1.32 | 3.67±0.83 |
| LP4 | 202.91±33.95 | 28.68±13.44 | 9.69±6.89 | 1.33±0.25 | 2.75±0.45 | 0.11±0.01 | 1.39±0.24 | 4.21±0.21 | 0.55±0.05 | 18.94±2.40 | 1.78±0.29 |
| LP5 | 394.65±27.23 | 56.57±14.71 | 5.67±2.36 | 1.05±0.06 | 2.43±0.19 | 0.09±0.01 | 1.32±0.18 | 7.02±1.13 | 0.57±0.15 | 17.70±4.33 | 2.67±0.37 |
| LP6 | 82.59±0.00 | 8.86±0.00 | 9.50±4.20 | 0.85±0.21 | 2.19±0.17 | 0.09±0.02 | 4.76±0.64 | 39.65±10.45 | 0.58±0.10 | 17.26±3.12 | 2.42±0.82 |
| LP7 | 136.06±45.25 | 20.04±17.06 | 2.12±0.10 | 0.53±0.06 | 1.30±0.09 | 0.06±0.01 | 0.90±0.12 | 7.04±1.00 | 0.34±0.04 | 17.19±0.91 | 3.46±0.98 |
| LP8 | 111.17±11.63 | 19.40±9.42 | 12.03±6.05 | 0.66±0.04 | 1.65±0.06 | 0.06±0.01 | 1.65±0.32 | 2.43±0.54 | 0.46±0.01 | 18.47±0.82 | 5.96±0.50 |
| LP9 | 74.43±29.69 | 11.62±7.61 | 10.14±3.21 | 0.38±0.04 | 1.11±0.22 | 0.04±0.01 | 1.36±0.49 | 5.72±2.24 | 0.35±0.05 | 17.06±0.55 | 1.89±0.85 |
| LP10 | 204.28±8.13 | 28.43±4.00 | 28.18±14.36 | 0.49±0.10 | 1.56±0.16 | 0.07±0.01 | 1.17±0.61 | 13.28±2.21 | 0.65±0.14 | 19.75±1.43 | 0.30±0.38 |
| IMG1 | 126.23±27.88 | 34.03±25.64 | 27.41±7.90 | 0.59±0.11 | 0.88±0.11 | 0.07±0.01 | 0.84±0.22 | 24.20±3.91 | 0.29±0.06 | 27.75±2.65 | 7.21±2.25 |
| IMG2 | 110.26±39.39 | 37.60±16.64 | 9.22±0.53 | 0.77±0.00 | 0.82±0.06 | 0.09±0.01 | 0.77±0.09 | 25.07±0.37 | 0.14±0.01 | 24.61±0.38 | 1.15±0.25 |
| IMG3 | 258.94±24.00 | 63.44±27.68 | 29.85±2.30 | 2.87±0.11 | 2.65±0.19 | 0.25±0.02 | 1.79±0.08 | 28.65±7.66 | 0.42±0.03 | 20.66±1.13 | 6.49±0.52 |
| IMG4 | 123.87±29.52 | 36.64±24.45 | 9.33±4.41 | 1.50±0.09 | 1.43±0.06 | 0.14±0.01 | 1.06±0.07 | 47.19±5.59 | 0.27±0.03 | 24.49±1.77 | 0.33±0.05 |
| IMG5 | 79.71±12.88 | 6.14±3.53 | 10.98±6.08 | 1.74±0.06 | 1.67±0.08 | 0.18±0.01 | 4.16±0.72 | 13.63±6.37 | 0.50±0.04 | 33.12±0.78 | 0.56±0.15 |
| IMG6 | 38.71±8.67 | 19.12±6.36 | 8.83±3.98 | 2.05±0.06 | 2.14±0.32 | 0.23±0.04 | 1.98±0.93 | 26.88±20.02 | 0.56±0.08 | 32.53±2.2 | 6.88±1.14 |
| IMG7 | 137.96±57.90 | 38.12±27.29 | 31.19±7.14 | 2.32±0.07 | 2.16±0.32 | 0.22±0.03 | 1.36±0.17 | 53.74±4.63 | 0.36±0.02 | 22.22±1.35 | 6.54±2.23 |
| IMG8 | 86.39±7.62 | 27.79±4.08 | 6.04±3.62 | 1.33±0.07 | 0.86±0.05 | 0.10±0.01 | 0.78±0.14 | 22.09±4.04 | 0.27±0.03 | 23.54±1.12 | 4.43±1.01 |
| IMG9 | 25.51±6.85 | 14.90±15.67 | 7.39±1.06 | 0.68±0.07 | 0.44±0.05 | 0.05±0.01 | 0.55±0.08 | 15.44±5.67 | 0.23±0.04 | 22.89±11.36 | 4.18±0.48 |
| IMG10 | 16.06±5.68 | 6.10±3.56 | 4.05±0.65 | 0.29±0.04 | 0.20±0.05 | 0.03±0.01 | 0.44±0.10 | 5.08±0.90 | 0.17±0.02 | 33.51±1.85 | 3.63±1.71 |
| QTP1 | 334.40±95.10 | 82.52±31.21 | 44.16±10.97 | 5.16±1.24 | 4.34±1.44 | 0.41±0.12 | 4.80±0.80 | 54.27±25.55 | 0.55±0.16 | 13.49±1.64 | 7.22±2.67 |
| QTP2 | 108.96±49.87 | 250.27±186.21 | 50.40±4.31 | 4.94±0.85 | 4.63±0.26 | 0.41±0.05 | 4.18±0.26 | 31.50±20.95 | 0.76±0.12 | 19.81±0.43 | 6.92±2.28 |
| QTP3 | 156.34±29.81 | 49.99±3.15 | 15.35±2.66 | 4.61±1.23 | 3.86±0.64 | 0.32±0.05 | 2.79±0.57 | 27.68±14.69 | 0.53±0.02 | 17.51±0.70 | 6.77±0.96 |
| QTP4 | 23.61±0.68 | 12.54±8.92 | 19.11±1.99 | 0.61±0.09 | 0.84±0.13 | 0.09±0.02 | 0.95±0.17 | 11.80±1.97 | 0.23±0.03 | 15.08±1.44 | 2.91±0.92 |
| QTP5 | 25.15±4.34 | 22.99±8.82 | 21.57±4.10 | 1.10±0.08 | 2.15±0.24 | 0.13±0.00 | 1.03±0.14 | 18.93±6.64 | 0.26±0.02 | 18.79±1.89 | 4.85±1.68 |
| QTP6 | 20.32±2.47 | 13.26±14.80 | 14.91±7.15 | 0.74±0.08 | 1.72±0.04 | 0.07±0.00 | 0.76±0.22 | 12.01±2.48 | 0.24±0.13 | 13.41±6.71 | 2.27±0.79 |
| QTP7 | 66.14±38.29 | 22.04±11.77 | 20.51±1.17 | 0.88±0.10 | 4.11±0.36 | 0.11±0.02 | 1.66±0.36 | 25.98±6.18 | 0.38±0.04 | 17.28±2.30 | 3.79±1.55 |
| QTP8 | 25.34±4.63 | 6.24±8.22 | 7.81±5.92 | 0.75±0.00 | 0.87±0.07 | 0.09±0.01 | 2.31±0.21 | 8.36±10.27 | 0.84±0.06 | 20.57±0.53 | 3.33±0.87 |
| QTP9 | 15.22±5.28 | 9.71±4.92 | 5.50±1.36 | 0.52±0.07 | 0.63±0.08 | 0.03±0.00 | 0.88±0.28 | 1.35±0.18 | 0.34±0.11 | 25.71±9.81 | 4.59±1.40 |
| QTP10 | 17.06±3.38 | 3.14±2.24 | 8.51±1.49 | 0.39±0.04 | 0.33±0.04 | 0.04±0.01 | 0.47±0.18 | 1.35±0.41 | 0.41±0.30 | 17.52±13.3 | 4.99±0.73 |

PC: Plant coverage (%), AGB: Aboveground biomass (g m^-2^), RB: Root biomass (kg m^-2^), PLSH: Plant simpson diversity (unitless), pH (unitless), CON: conductivity (μs cm^-1^), BD: Bulk density (g cm^-3^), Clay (%), Silt (%), Sand (%), MBC: Microbial biomass carbon (mg kg^-1^), MBN: Microbial biomass nitrogen (mg kg^-1^), MBP: Microbial biomass phosphorus (mg kg^-1^), TOC: Total organic carbon (%), TC: Total carbon (%), TN: Total nitrogen (%), NH_4_^+^-N (mg kg^-1^), NO_3_^-^-N (mg kg^-1^), TP: Total phosphorus (g kg^-1^), TK: Total potassium (g kg^-1^), AP: Available phosphorus (mg kg^-1^). Values are expressed as the mean with a standard error (SE).

Table S3 Information of biotic interactions across Loess plateau (LP), Inner Mongolian (IMG) and Qinghai- Tibet Plateau (QTP) transects.

| Plots ID | No.Nodes | No.Edges | No.Modules | Average.Degree | Avg.path.length | Clust.Coefficient | Connectivity | Modularity |
| --- | --- | --- | --- | --- | --- | --- | --- | --- |
| LP1 | 22±8.29 | 33.50±10.25 | 3.50±1.73 | 3.11±0.27 | 2.28±0.35 | 0.41±0.05 | 0.17±0.08 | 0.34±0.11 |
| LP2 | 10±2.35 | 10.00±1.73 | 3.00±1.00 | 2.04±0.23 | 2.23±0.53 | 0.65±0.09 | 0.25±0.12 | 0.27±0.14 |
| LP3 | 13.6±6.31 | 12.00±8.72 | 4.20±1.10 | 1.61±0.44 | 1.73±0.29 | 0.31±0.29 | 0.14±0.03 | 0.54±0.03 |
| LP4 | 9.00±1.73 | 5.67±3.06 | 4.67±1.15 | 1.22±0.51 | 1.47±0.17 | 0.45±0.40 | 0.15±0.05 | 0.22±0.19 |
| LP5 | 24.6±11.41 | 32.40±24.01 | 5.20±1.30 | 2.27±1.03 | 2.47±0.69 | 0.34±0.07 | 0.10±0.01 | 0.40±0.08 |
| LP6 | 30.6±4.56 | 47.80±5.85 | 7.00±1.87 | 3.17±0.53 | 2.5±0.24 | 0.44±0.03 | 0.11±0.04 | 0.42±0.03 |
| LP7 | 27.6±1.16 | 40.3±7.05 | 6.10±2.20 | 2.99±0.83 | 2.49±0.29 | 0.40±0.06 | 0.1±0.03 | 0.41±0.05 |
| LP8 | 21.75±4.43 | 176.75±65.81 | 2.00±0.00 | 15.81±2.90 | 1.12±0.01 | 0.94±0.01 | 0.77±0.03 | 0.02±0.01 |
| LP9 | 24.2±5.07 | 173.20±58.50 | 4.00±1.22 | 13.93±2.55 | 1.1±0.04 | 0.94±0.02 | 0.61±0.06 | 0.02±0.01 |
| LP10 | 22.3±4.37 | 174.7±61.05 | 3.5±1.09 | 14.73±2.61 | 1.11±0.05 | 0.93±0.03 | 0.65±0.05 | 0.02±0.02 |
| IMG1 | 0.67±1.15 | 0.33±0.58 | 0.33±0.58 | 0.33±0.58 | 0.33±0.58 | 0.00±0.00 | 0.33±0.58 | 0.00±0.00 |
| IMG2 | 4.00±1.00 | 1.00±0.00 | 3.00±1.00 | 0.53±0.13 | 1.00±0.00 | 0.00±0.00 | 0.22±0.12 | 0.00±0.00 |
| IMG3 | 6.00±5.00 | 2.00±2.00 | 4.00±3.00 | 0.36±0.36 | 0.60±0.60 | 0.00±0.00 | 0.04±0.04 | 0.31±0.31 |
| IMG4 | 9.8±0.89 | 6±2.44 | 7.80±1.40 | 0.56±0.32 | 1.41±0.67 | 0.00±0.00 | 0.12±0.06 | 0.00±0.00 |
| IMG5 | 12.8±1.64 | 8±0.79 | 9.30±1.50 | 1.67±0.97 | 2.81±0.88 | 0.12±0.02 | 0.24±0.03 | 0.40±0.03 |
| IMG6 | 5.80±0.84 | 1.00±0.71 | 4.80±1.10 | 0.35±0.29 | 0.87±0.51 | 0.00±0.00 | 0.08±0.07 | 0.00±0.00 |
| IMG7 | 14.75±13.65 | 8.00±9.06 | 7.75±6.55 | 0.74±0.53 | 1.38±1.08 | 0.09±0.18 | 0.05±0.03 | 0.47±0.32 |
| IMG8 | 16.00±7.81 | 23.00±18.03 | 5.00±1.00 | 2.43±1.42 | 1.70±0.39 | 0.36±0.32 | 0.16±0.02 | 0.30±0.13 |
| IMG9 | 14.00±7.31 | 26.00±12.21 | 2.80±3.49 | 3.84±1.37 | 1.77±0.25 | 0.51±0.21 | 0.35±0.19 | 0.16±0.08 |
| IMG10 | 16.00±2.58 | 37.50±8.39 | 2.25±0.96 | 4.67±0.67 | 1.78±0.17 | 0.53±0.05 | 0.32±0.06 | 0.15±0.03 |
| QTP1 | 4.25±1.26 | 1.50±0.58 | 2.75±0.96 | 0.71±0.21 | 1.00±0.00 | 0.00±0.00 | 0.24±0.11 | 0.25±0.29 |
| QTP2 | 4.25±0.50 | 2.00±0.00 | 2.25±0.50 | 0.95±0.10 | 1.00±0.00 | 0.00±0.00 | 0.30±0.07 | 0.50±0.00 |
| QTP3 | 3.50±3.79 | 4.25±7.85 | 1.50±0.58 | 1.06±1.70 | 0.57±0.67 | 0.19±0.39 | 0.19±0.23 | 0.04±0.08 |
| QTP4 | 37.00±7.12 | 204.50±60.87 | 4.00±1.15 | 10.86±1.68 | 2.00±0.20 | 0.70±0.02 | 0.31±0.04 | 0.19±0.01 |
| QTP5 | 40.40±2.88 | 260.40±17.29 | 3.60±0.55 | 12.92±0.97 | 1.81±0.11 | 0.68±0.01 | 0.33±0.04 | 0.18±0.02 |
| QTP6 | 23.00±9.70 | 61.20±38.15 | 4.20±1.48 | 4.91±1.44 | 2.01±0.54 | 0.67±0.07 | 0.25±0.10 | 0.31±0.09 |
| QTP7 | 15.33±5.03 | 37.00±16.52 | 3.67±1.15 | 4.70±1.21 | 1.55±0.41 | 0.70±0.11 | 0.35±0.09 | 0.16±0.13 |
| QTP8 | 4.67±2.31 | 2.33±2.08 | 2.33±0.58 | 0.78±0.69 | 1.10±0.96 | 0.00±0.00 | 0.16±0.14 | 0.07±0.13 |
| QTP9 | 16.25±6.34 | 18.75±11.44 | 4.25±1.50 | 2.18±0.53 | 2.49±0.83 | 0.59±0.1 | 0.16±0.06 | 0.40±0.17 |
| QTP10 | 5.33±3.79 | 7.67±8.02 | 2.00±1.00 | 2.00±2.00 | 0.75±0.66 | 0.64±0.56 | 0.30±0.29 | 0.09±0.13 |
